# Soluble Epoxide Hydrolase and Brain Cholesterol Metabolism

**DOI:** 10.3389/fnmol.2019.00325

**Published:** 2020-01-29

**Authors:** Michelle Flores Domingues, Natalia Callai-Silva, Angela Regina Piovesan, Celia Regina Carlini

**Affiliations:** ^1^Graduate Program in Cellular and Molecular Biology, Center of Biotechnology, Universidade Federal do Rio Grande do Sul, UFRGS, Porto Alegre, Brazil; ^2^Laboratory of Neurotoxins, Brain Institute (BRAINS-InsCer), Pontifícia Universidade Católica do Rio Grande do Sul, Porto Alegre, Brazil; ^3^Graduate Program in Medicine and Health Sciences, Pontifícia Universidade Católica do Rio Grande do Sul, Porto Alegre, Brazil

**Keywords:** soluble epoxide hydrolase, N-terminal domain, phosphatase activity, brain cholesterol metabolism, Alzheimer’s disease

## Abstract

The bifunctional enzyme soluble epoxide hydrolase (sEH) is found in all regions of the brain. It has two different catalytic activities, each assigned to one of its terminal domains: the C-terminal domain presents hydrolase activity, whereas the N-terminal domain exhibits phosphatase activity. The enzyme’s C-terminal domain has been linked to cardiovascular protective and anti-inflammatory effects. Cholesterol-related disorders have been associated with sEH, which plays an important role in the metabolism of cholesterol precursors. The role of sEH’s phosphatase activity has been so far poorly investigated in the context of the central nervous system physiology. Given that brain cholesterol disturbances play a role in the onset of Alzheimer’s disease (AD) as well as of other neurodegenerative diseases, understanding the functions of this enzyme could provide pivotal information on the pathophysiology of these conditions. Moreover, the sEH phosphatase domain could represent an underexplored target for drug design and therapeutic strategies to improve symptoms related to neurodegenerative diseases. This review discusses the function of sEH in mammals and its protein structure and catalytic activities. Particular attention was given to the distribution and expression of sEH in the human brain, deepening into the enzyme’s phosphatase activity and its participation in brain cholesterol synthesis. Finally, this review focused on the metabolism of cholesterol and its association with AD.

## Introduction

Cholesterol metabolism in the brain is independent of peripheral tissues due to the blood-brain barrier (BBB) that impairs the entrance of the protein-bound lipid into the central nervous system (CNS). Brain tissues contain large amounts of cholesterol, up to 25% of the body’s cholesterol content (Dietschy, 2009) whose metabolism and complex homeostasis regulation in the CNS remain unclear (Zhang and Liu, 2015). Changes in this metabolism are related to neurodegenerative pathologies, such as Alzheimer’s disease (AD), Parkinson’s disease, and Huntington’s disease, as well as to age-related cognitive decline (Petrov et al., 2016; Loera-Valencia et al., 2019).

Soluble epoxide hydrolase (sEH), a bifunctional enzyme possessing two different catalytic activities, is found in all regions of the brain (Sura et al., 2008). The C-terminal domain of its polypeptide chain exhibits EH activity, whereas the N-terminal domain presents phosphatase activity (Morisseau et al., 2012). The role of sEH’s C-terminal domain has been extensively investigated (reviewed in Imig, 2013; Morisseau and Hammock, 2013; He et al., 2016), but the role of its N-terminal domain is still poorly understood (see Table 1). Endogenous substrates for sEH N-terminal activity appear to be phosphorylated lipids, some of which are precursors in the cholesterol biosynthesis pathway and for protein isoprenylation (Enayetallah and Grant, 2006), as well as lysophosphatidic acids and sphyngosine-1-phosphate (Oguro et al., 2009; Morisseau et al., 2012). Considering the abundance of cholesterol in the CNS, it is important to further investigate the role of sEH in the brain cholesterol pathway.

**TABLE 1:** Summary of studies investigating the roles of the carboxy- and the amino terminal domains of the soluble epoxide hydrolase enzyme.

Soluble epoxide hydrolase	Effects	Experimental models	References
**C-terminal domain (hydrolase activity)**	Expression of human sEH domain alone reduces cholesterol levels.	Cell culture of HepG2 cell line and sEH-knockout mice	EnayetAllah et al., 2008
	C-terminal inhibitor promotes neuroprotective effects, such as attenuation of oxidative stress, apoptosis, protein aggregation, and endoplasmic reticulum stress.	C57BL/6J mice MPTP-induced neurotoxicity	Huang et al., 2018
	sEH inhibition protects neurons and suppresses cytokine production and microglial migration into the hippocampus.	Male C57BL/6 mice global cerebral ischemia-induced	Taguchi et al., 2016
	C-terminal inhibition promotes antidepressant effect, increase of hippocampal BDNF expression, and neurogenesis.	Male C57BL/6 mice	Wu et al., 2017
	Inhibition of C-domain increases the memory response, reduces oxidative stress, minimizes inflammation, and decreases the level of the amyloid precursor protein in the brain.	Male Sprague-Dawley rats streptozotocin-induced Alzheimer’s disease-like	Pardeshi et al., 2019
	Inhibition of C-terminal domain enhanced neuronal synaptic neurotransmission in the PFC associated with enhanced postsynaptic glutamatergic receptor and long-term potentiation (LTP) *via* extracellular signal-regulated kinase (ERK) phosphorylation.	Brain slices of C57BL/6 mice	Wu et al., 2015
**N-terminal domain (phosphatase activity)**	Negatively regulates eNOS activity and NO production	Cell culture of bovine aortic endothelial cells and sEH-knockout mice	Hou et al., 2011, 2015
	Expression of human sEH phosphatase domain alone increases cholesterol levels.	Cell culture of HepG2 cell line and sEH-knockout mice	EnayetAllah et al., 2008
	Arg287Gln variant is linked to increased levels of plasma cholesterol and of triglycerides in patients with familial hypercholesterolemia.	Human blood plasma	Sato et al., 2004

This review aims to summarize the role of sEH in mammals and its protein structure and catalytic activities. We also provide a compilation of sEH distribution and expression in the human brain and deepen the discussion on the enzyme’s N-terminal phosphatase activity and its involvement in the synthesis of cholesterol. Finally, we discuss cholesterol metabolism and its implication in AD.

## Soluble Epoxide Hydrolase

Vertebrate sEHs are members of the EH family ubiquitously found in nature (Enayetallah et al., 2004; Newman et al., 2005; Morisseau and Hammock, 2013). EHs open epoxides to form diols by the addition of a water molecule (Fretland and Omiecinski, 2000). In mammals, this enzyme is broadly distributed in different tissues. The subcellular localization of sEH follows a tissue-specific pattern, by which the protein can be exclusively located in the cytosol or additionally present in peroxisomes (Enayetallah et al., 2004; Newman et al., 2005; Enayetallah and Grant, 2006; Kramer and Proschak, 2017). Mammals have several EH isoforms, the most known of which are the microsomal EH (mEH) and sEH (Decker et al., 2009). Two other EHs have been described, EH3 and EH4, and they represent a new family of mammalian EHs. EH3 is mostly expressed in the lung, skin, and upper gastrointestinal tract and features a high activity for fatty acid-derived epoxides (Decker et al., 2012).

In humans, the bifunctional enzyme is located intracellularly, both in the cytosol and in peroxisomes (Newman et al., 2005), with a broad distribution in all tissues (Enayetallah et al., 2004; Sura et al., 2008). A function for sEH was first described in the metabolism of xenobiotic compounds by the kidney and the liver (Decker et al., 2009). While the detoxifying role of hepatic and renal sEH has been carefully addressed (Imig and Hammock, 2009; Imig, 2013), much less is known about the role of the sEH in the brain.

## Distribution and Expression of sEH in the Brain

In the human CNS, the sEH is distributed in all regions of the brain, mostly in neuronal cell bodies, as well as in astrocytes and oligodendrocytes. It also occurs in a relatively high abundance in the ependymal cells of the choroid plexus and in the smooth muscle of brain arterioles (Sura et al., 2008). In the brain of mice, immunoreactivity for sEH was found only in neurons of the central amygdala, which also contained mEH. Rather than in neurons, in other CNS regions, sEH was located in astrocytes (Marowsky et al., 2009; Harris and Hammock, 2013). Neurons in the nucleus, which represent an important output of the amygdala, express a number of neuropeptides, the release of which are thought to recruit BK_*Ca*_ channels for calcium signaling, in most neuronal secretory cells (Cassell and Gray, 1989). Epoxyeicosatrienoic acids (EETs) and/or dihydroxyeicosatrienoic acids (DHETs) could play a role on neuropeptide release because of their well-known actions on BK_*Ca*_ channels. Accordingly, it has been shown that 14,15-EET has a role in the release of oxytocin and vasopressin (Negro-Vilar et al., 1985), somatostatin (Junier et al., 1990), and β-endorphin and Met-enkephalin (Terashvili et al., 2008). Altogether, it is possible that the exclusive neuronal presence of sEH in the central amygdala might have a function in the release of neuropeptides.

Blockage of sEH activity, either through pharmacological inhibition or genetic deletion, revealed its neuroprotective effects. In sEH knockout mice, decreased infarct volumes, elevated brain-derived neurotrophic factor (BDNF) expression, and increased production of astrocyte-derived BDNF were reported, all favoring astrocyte viability and protecting neurons from ischemic injury (Yuan et al., 2016). sEH activity affects basal synaptic transmission and synaptic plasticity. Furthermore, its inhibition improves neuronal synaptic neurotransmission mediated by an increase of postsynaptic glutamatergic receptors and long-term potentiation, which is boosted *via* extracellular signal-regulated kinase (ERK) phosphorylation (Wu et al., 2015), suggesting that inhibition of sEH could modulate learning and memory formation. sEH inhibitors have thus been proposed as potential therapeutic agents for the prevention and treatment of injuries to the CNS.

### sEH Protein Structure

**FIGURE 1:**
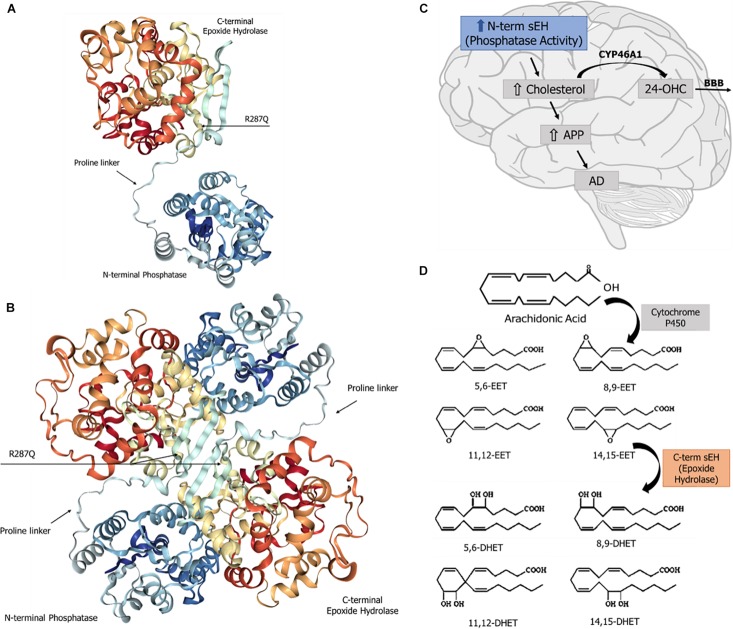
Soluble epoxide hydrolase (sEH): structure and known physiological roles. **(A)** Structure of sEH’s monomer, with the N- and C-terminal domains separated by a proline-rich linker. The SNP R287Q, located in the C-terminal domain, results in a lower hydrolase activity. **(B)** Overall structure of the dimeric form of the human sEH, showing the two monomers and their respective domains N- and C-termini, which are globular regions with alpha and beta structures connected by a proline-rich linker. The amino terminal domain displays a phosphatase activity, whereas the carboxy terminal domain carries a hydrolase activity. **(C)** The sEH’s phosphatase activity is involved in brain cholesterol metabolism. Higher brain cholesterol content may increase the levels of amyloid precursor protein (APP), thus contributing to the pathogenesis of Alzheimer’s disease (AD). **(D)** The sEH activity performs the hydration of epoxide groups of all six regioisomers of neuroprotective epoxyeicosatrienoic acids (EET), forming the corresponding inactive dihydrodiols.

The human sEH is a 62.5-kDa enzyme, product of the EPXH2 gene, located on chromosome 8. This gene (45 kb) contains 19 exons encoding a protein of 555 amino acid residues (Morisseau and Hammock, 2013) and encodes a two-domain, bifunctional enzyme as result of a gene fusion event (Beetham et al., 1995). In mammals, sEH is a homodimer composed of two globular monomers, where each monomer comprises two distinct structural domains, the N- and C-terminal regions, separated by a short proline-rich linker (Newman et al., 2005). The dimer shows an “antiparallel” organization of the monomers, in such a way that the C-terminal region of one monomer faces the N-terminal region of the other. The structural features of sEH are depicted in Figure 1. The C-terminal domain (EC 3.3.2.10), ∼35 kDa, exhibits EH activity, which opens an epoxide ring to form the corresponding diol. On the other hand, the N-terminal domain (EC 3.1.3.76), ∼25 kDa, has phosphatase activity toward lipid phosphates, such as isoprenoid phosphates, lysophosphatidic acids, and sphingosine-1-phosphate (Harris and Hammock, 2013; Morisseau and Hammock, 2013; Morisseau et al., 2013). The domain-swapped architecture of sEH aids in the enzyme’s stability and enables exchange of two distinct unrelated substrates (Argiriadi et al., 1999). The combined phosphatase and EH domains in this bifunctional enzyme reflects a positive selective advantage of a gene fusion event between ancient members of two distinct superfamilies of dehalogenases. Single-domain EHs are found in plants, invertebrates, and fungi and show homology to prokaryotic haloalkane dehalogenase (Beetham et al., 1995; Harris et al., 2008). The activity of the N-terminal domain of the first “fused” enzymes was only vestigial (Beetham et al., 1995) and evolved through mutations to the present phosphatase domain of vertebrate sEH, whose enzyme activity was discovered only in 2003 (Cronin et al., 2003; Newman et al., 2005). Orthologs of the vertebrate sEH phosphatase domain were described in the genome of *Caenorhabditis elegans* as well as in Archea and Bacteria (Harris et al., 2008; Harris and Hammock, 2013).

## Catalytic Activities of sEH

### Epoxide Hydrolase

The sEH C-terminal EH domain metabolizes all four regioisomers of EET, which are lipid autacoids derived from arachidonic acid by the action of cytochrome P450 epoxygenases. sEH introduces a water molecule into epoxide ring to form the corresponding DHET (Figure 1), thereby diminishing or eliminating the biological effects of EET (Yu et al., 2000).

Studies with potent selective inhibitors, such as 1-trifluoromethoxyphenyl-3-(1-propionylpiperidin-4-yl) urea, N-[1-(1-Oxopropyl)-4-piperidinyl]-N′-[4-(trifluoromethoxy)ph enyl] urea (TPPU), or 12-[[(tricyclo [3.3.1.13,7] dec-1-ylamino) carbonyl] amino]-dodecanoic acid (AUDA), have indicated that inhibition of sEH activity promotes anti-inflammatory, antihypertensive, neuroprotective, and cardioprotective effects (Shen and Hammock, 2012). These beneficial effects correlated to an increase of the circulating levels of EET, which contribute to halt the progression of hypertension, cardiovascular pathologies, hypercholesterolemia, and of inflammatory diseases (Thomson et al., 2012).

### Phosphatase Activity

The physiological role and the endogenous substrate(s) of the sEH phosphatase activity are still elusive. Studies performed *in vitro* indicated that sEH phosphatase activity hydrolyzes lipid phosphates, like isoprenoid phosphates, which are precursors of cholesterol biosynthesis and protein isoprenylation (Enayetallah and Grant, 2006). Morisseau and collaborators (Morisseau et al., 2012) have demonstrated that lysophosphatidic acids (LPAs) are excellent substrates for sEH phosphatase activity. LPAs bind to various nuclear receptors and regulate cell survival, apoptosis, motility, shape, differentiation, gene transcription, and malignant transformation (Sciorra and Morris, 2002; Lin et al., 2010). Moreover, LPA inhibits pre-adipocyte differentiation, thus limiting adipogenesis through interaction with PPARγ (Morris et al., 2009). This nuclear receptor plays an essential role in regulating lipid and glucose homeostasis.

Several groups have shown that fatty acid epoxide substrates and diol products of sEH activate peroxisome proliferator-activated receptors (PPARs) (Oguro et al., 2009; Iyer et al., 2011). The exact role of sEH in modulating PPARs is currently unknown, albeit the significance of PPARs in lipid metabolism and metabolic disorders is well-documented (Lee et al., 2003; Cho et al., 2008).

Soluble epoxide hydrolase also plays a pivotal role in the regulation of endothelial nitric oxide (NO) synthase (eNOS) activity and NO-mediated endothelial cell functions (Hou et al., 2011). Endothelium-derived NO is a crucial regulator of vascular homeostasis (Schulz et al., 2008). NO bioavailability is tightly controlled by NOS activity. The phosphatase activity of sEH downregulates activated eNOS signaling and NO production. Inhibition of sEH phosphatase activity was shown to increase NO production and prolong the phosphorylation of eNOS (Hou et al., 2011; Hou et al., 2015). In the nervous system, NO has both physiological and pathological functions. For example, NO contributes to long-term potentiation (LTP), playing a role in learning and memory processes (Schuman and Madison, 1991). NO enhances the expression of cAMP response element binding protein (CREB) to mediate the response to BDNF (Riccio et al., 2006). NO also mediates glutamate-NMDA receptor (NMDAr) signaling in neurons (Jafari et al., 2018; Wu and Tymianski, 2018).

To date, different molecules with inhibitory effect toward the phosphatase activity of sEH have been described, even though most lack a potent and selective chemical action. Kramer and collaborators reported (Kramer et al., 2019) in 2019 the synthesis of the first *in vivo* inhibitor of both the human and rat sEH phosphatase domains, 4-(4-(3,4-dichlorophenyl)-5-phenyloxazol-2-yl) butanoic acid (SWE101), with an IC_50_ 0.058 μM against the human enzyme. SWE101 showed adequate pharmacokinetic properties in rats, allowing studies on the pathophysiological relevance of phosphatase activity of sEH and revealing its role on the hydrolysis of lysophosphatidic acids (Kramer and Proschak, 2017; Kramer et al., 2019).

## sEH Phosphatase Activity and Synthesis of Cholesterol

Epidemiological studies have associated sEH polymorphisms with various human diseases, many of which are related to cardiovascular disorders and familial hypercholesterolemia (Sato et al., 2004). The R287Q variant was described as an emerging risk factor for atherosclerosis. This polymorphism correlated positively with subclinical atherosclerosis and coronary artery calcification (Fornage et al., 2004; Wei et al., 2007). The R287Q variant is also linked to increased levels of plasma cholesterol and triglycerides in patients with familial hypercholesterolemia (Sato et al., 2004). This sEH variant displays a higher isoprenoid phosphatase activity *in vitro* (Enayetallah and Grant, 2006). Another variant, sEH K55R, leads to an increased sEH activity *in vivo* and was correlated with coronary heart disease (Lee et al., 2006). The single-nucleotide polymorphisms (SNPs) cited above occur in two different exons of the EPHX2 gene. The former, located in the eighth exon, results in a change of the amino acid (arginine to glutamine) in position 287 in the C-terminal domain and a lower hydrolase activity. The latter SNP resides in the second exon, encoding an amino acid substitution (lysine to arginine) in the N-terminal domain of the polypeptide chain and causes an increase of the hydrolase activity (Przybyla-Zawislak et al., 2003).

Soluble epoxide hydrolase-knockout mice have lower cholesterol levels as well as several of its precursors, besides reduced plasma steroid concentration. This was accompanied by a general decrease in serum lipids (EnayetAllah et al., 2008; Luria et al., 2009). Studies with recombinant HepG2 cells expressing either the phosphatase domain or the hydrolase domain alone have demonstrated the independent and opposite roles of the two sEH enzymatic activities. The C-terminal activity lowered the cell’s cholesterol level while the N-terminal activity was found to increase it (EnayetAllah et al., 2008). Treatment of wild-type mice with a hydrolase inhibitor resulted in higher cholesterol levels, thus mimicking the effect of expressing the phosphatase domain alone in HepG2 cells (EnayetAllah et al., 2008).

Besides its direct catalytic activity on so far unidentified substrate(s), sEH further affects cholesterol metabolism by modulating the expression of the rate-limiting enzyme in cholesterol synthesis, HMG CoA reductase, *via* the activation of AMPK and inhibition of SREBP1/2 (Mangels et al., 2016). Moreover, sEH deletion also affected the basal and insulin-induced expressions of low-density lipoprotein (LDL) receptor and of fatty acid synthase (Mangels et al., 2016).

Altogether, these results suggest a potential role of sEH in regulating cholesterol biosynthesis and metabolism.

## Brain Cholesterol Metabolism

Cholesterol is an essential molecule for the neuronal physiology during early development as well as in the adult life, and its levels must be precisely maintained to allow for its correct function. Both neurons and glial cells produce enough cholesterol for their survival. Cholesterol is necessary for the growth of axons and dendrites during organogenesis, nerve regeneration, remodeling, and neuronal repair (Boyles et al., 1989; de Chaves et al., 1997; Mahley and Rall, 2000). Additional amounts are required for the formation of mature synapses, and synaptogenesis might be limited by the availability of cholesterol (Mauch et al., 2001).

Cholesterol synthesis and metabolism in the brain fully rely on *de novo* synthesis. The BBB, which prevents cholesterol uptake from the circulation, and the blood-cerebrospinal fluid (CSF) barrier (plasma is ultrafiltrated to be part of the CSF) are two natural barriers that prevent mixing of plasma and brain cholesterol (Gamba et al., 2019; Genaro-Mattos et al., 2019).

Similar to peripheral cholesterol synthesis, brain cholesterol is synthesized from acetyl-CoA in a complex biosynthetic pathway catalyzed by at least 20 enzymes, including the rate-limiting HMG-CoA reductase (Gamba et al., 2019). In glial cells, brain cholesterol is converted mainly into 24-hydroxycholesterol (24-OHC) or oxidized into 27-hydroxycholesterol (27-OHC). The latter regulates cholesterol transport in neurons by acting on a nuclear transcription factor that controls the expression of ApoE and ABCA1/ABCG1 (Czuba et al., 2017). Such oxysterols are important to maintain brain cholesterol homeostasis.

The expression levels of cholesterol 24-hydroxylase, a cytochrome P450 enzyme (CYP46A1), is about 100-fold higher in the brain than in the liver. This enzyme catalyzes the conversion of CNS cholesterol to 24-OHC, an oxidized catabolite denominated oxysterol. Most of the oxysterol flows out from the brain into the blood through the BBB, powered by the concentration gradient, while a small amount flows into the CSF (Lütjohann et al., 1996; Björkhem et al., 2018). In contrast, the catabolic pathway of cholesterol *via* oxidation in the liver has as final products the bile acids (Lund et al., 2003).

To evaluate the importance of the 24-hydroxylation pathway in cholesterol turnover, Lund and collaborators (Lund et al., 2003) conducted studies in cholesterol 24-hydroxylase knockout mice (CYP46A1_/_), showing that *de novo* cholesterol synthesis in the liver remained stable, whereas that in the brain was decreased by 40%. These data indicated that *de novo* cholesterol synthesis and the levels of 24-OHC are strongly linked, and that at least 40% of cholesterol turnover in the brain depends on cholesterol 24-hydroxylase. Thus, this enzyme constitutes an important tissue-specific pathway for cholesterol renewal in the brain.

24-hydroxycholesterol plasma levels, unlike other oxysterols, show an age-dependent correlation: in the first decade of life, the ratio between 24-OHC and cholesterol is about five times higher than it is in the sixth decade. Moreover, there is also a correlation between 24-OHC levels in the plasma and the CSF, indicating that 24-OHC efflux from the brain into the circulation is significant and necessary for cerebral cholesterol homeostasis (Lütjohann et al., 1996).

A direct correlation between a decrease in plasma cholesterol concentration and in HMG-CoA reductase was shown by EnayetAllah et al. (2008) in sEH-knockout male mice. Moreover, HepG2 cell lines subjected to inhibition of the sEH domain showed higher cholesterol levels. Altogether, these data suggest an involvement of sEH phosphatase domain in cholesterol metabolism. Thus, it is plausible to suggest that in the brain, a similar mechanism occurs, with the N-terminal domain of sEH regulating cholesterol metabolism, implying that higher levels of its phosphatase activity could potentially increase brain cholesterol concentrations.

## Alzheimer’s Disease and Cholesterol Metabolism

Alzheimer’s disease is the most common neurodegenerative disorder worldwide and also the major cause of dementia in the elderly. Despite extensive research, no cure for this pathology has been found. Classical hallmarks for AD include extracellular senile plaques formed by the deposition of β-amyloid peptide (Aβ), excessive phosphorylation of the tau protein, and formation of intracellular neurofibrillary tangles—all these processes leading to loss of synapses, neuronal death, and proliferation of astrocytes (Masters et al., 2015).

Hypercholesterolemia is an important risk factor for AD and other neurodegenerative diseases. Plasma cholesterol levels are at least 10% higher in AD patients (Popp et al., 2013), and those patients with higher levels of total cholesterol seem to have a faster decline in cognitive abilities than the ones with normal cholesterol levels (Helzner et al., 2009).

Disruptions in cholesterol metabolism are significantly associated with AD pathogenesis, even though it is uncertain if these are a cause or a consequence of the disease (Petrov et al., 2017). The efflux of oxysterols from the brain into the blood plays a key role in this process (Loera-Valencia et al., 2019). Genetic polymorphisms in proteins involved in energy metabolism or lipid transport are known as important risk factors for the development of this pathology, such as the presence of the allele ApoE4 of apolipoprotein E (ApoE), the most abundant apoprotein involved in cholesterol transport in the CNS (Holtzman et al., 2012). The presence of the ApoE4 allele is considered an important genetic risk factor for both early- and late-onset AD, accounting for 45–60% of AD patients (Farrer et al., 1997).

Increased cholesterol levels in the brain appear to contribute to changes in cell membrane properties consequent to the increased contents of intracellular and membrane-bound cholesterol (Xue-shan et al., 2016). The amyloid precursor protein (APP) is an integral transmembrane protein. When the amount of cholesterol in the cell membranes rises, it promotes the binding of APP to lipid rafts and its cleavage to form the β-amyloid peptides (Figure 1; Beel et al., 2010). There are multiple β-amyloid forms, such as Aβs monomers, oligomers, and amyloid fibers. Aβs oligomers are the most toxic and recognized as the primary cause of cognitive decline in AD patients (Viola and Klein, 2015). Furthermore, high intracellular cholesterol levels increase the enzymatic activity of secretases involved in the amyloid metabolic pathway, resulting in a higher generation of Aβ (Grimm et al., 2008).

The BBB plays an important role in limiting the entry of plasma components that are potentially neurotoxic, as well as pathogens and blood cells into the brain. Jiang and collaborators (Jiang et al., 2012) found that a high-cholesterol diet (2% cholesterol supplementation for 10 weeks) fed to rabbits increased the BBB permeability when compared to animals receiving a normal diet. This increased BBB permeability enables the peripheral cholesterol to cross this barrier, leading to further increase of the CNS cholesterol levels (Xue-shan et al., 2016). Thus, any dysfunction of the BBB can alter significantly the CNS environment, affecting its functions and structure, and plays a key role in the development and progression of AD.

Morisseau et al. (2013) discovered a compound, called ebselen, which inhibits sEH phosphatase activity in a high-throughput screening enzyme-based assay. Ebselen binds to the N-terminal domain of sEH and chemically reacts with the enzyme to quickly and irreversibly block its phosphatase activity (Morisseau et al., 2013). Noteworthy, Xie et al. (2017) reported in 2017 the potential of ebselen, a strong antioxidant, lipid-soluble, selenium-containing compound, in the treatment of cognitive dysfunction and neuropathology in a triple transgenic AD model in mice. Ebselen decreased the expression of amyloid precursor protein and β-secretase, reduced the levels of Aβ in AD neurons, especially the most toxic oligomeric form, and decreased phosphorylation of tau protein while increasing glutathione peroxidase and superoxide dismutase activities (Xie et al., 2017). The beneficial effects of ebselen were attributed solely to its antioxidant properties. However, these protective effects could also be, at least in part, related to the inhibition of sEH phosphatase activity and its consequent impact of lowering brain cholesterol levels.

## Conclusion

Throughout the years, human sEH has been poorly studied in the context of CNS physiology, mainly the role of its phosphatase domain and its possible influence on brain cholesterol metabolism. Considering that the disruption in brain cholesterol is a key player in the onset of AD, as well as of other neurodegenerative diseases, it is crucial to deepen our understanding of the role of this enzyme in the CNS pathophysiology. In that view, sEH phosphatase domain may represent an underexploited target for drug design and development of therapeutic strategies to ameliorate the symptoms or delay the progress of neurodegenerative diseases.

## Author Contributions

All authors reviewed the literature and wrote the manuscript.

## Conflict of Interest

The authors declare that the research was conducted in the absence of any commercial or financial relationships that could be construed as a potential conflict of interest.

## References

[B1] Argiriadi M. A. Morisseau C. Hammock B. D. Christianson D. W. (1999). Detoxification of environmental mutagens and carcinogens: structure, mechanism, and evolution of liver epoxide hydrolase. *Proc. Natl. Acad. Sci. U.S.A.* 96 10637–10642. 10.1073/pnas.96.19.10637 10485878 PMC17935

[B2] Beel A. J. Sakakura M. Barrett P. J. Sanders C. R. (2010). Direct binding of cholesterol to the amyloid precursor protein: an important interaction in lipid–Alzheimer’s disease relationships? *Bioch. Biophys. Acta Mol. Cell Biol. Lipids* 1801 975–982. 10.1016/j.bbalip.2010.03.008 20304095 PMC2886191

[B3] Beetham J. K. Grant D. Arand M. Garbarino J. Kiyosue T. Pinot F. (1995). Gene evolution of epoxide hydrolases and recommended nomenclature. *DNA Cell Biol.* 14 61–71. 10.1089/dna.1995.14.61 7832993

[B4] Björkhem I. Leoni V. Svenningsson P. (2018). On the fluxes of side-chain oxidized oxysterols across blood-brain and blood-CSF barriers and origin of these steroids in CSF. *J. Steroid Biochem. Mol. Biol.* 188 86–89. 10.1016/j.jsbmb.2018.12.009 30586624

[B5] Boyles J. K. Zoellner C. D. Anderson L. J. Kosik L. M. Pitas R. E. Weisgraber K. H. (1989). A role for apolipoprotein E, apolipoprotein AI, and low density lipoprotein receptors in cholesterol transport during regeneration and remyelination of the rat sciatic nerve. *J. Clin. Invest.* 83 1015–1031. 10.1172/jci113943 2493483 PMC303779

[B6] Cassell M. D. Gray T. S. (1989). Morphology of peptide-immunoreactive neurons in the rat central nucleus of the amygdala. *J. Comp. Neurol.* 281 320–333. 10.1002/cne.902810212 2468696

[B7] Cho M.-C. Lee K. Paik S. G. Yoon D. Y. (2008). Peroxisome proliferators-activated receptor (PPAR) modulators and metabolic disorders. *PPAR Res.* 2008:679137. 10.1155/2008/679137 18566691 PMC2430035

[B8] Cronin A. Mowbray S. Dürk H. Homburg S. Fleming I. Fisslthaler B. (2003). The N-terminal domain of mammalian soluble epoxide hydrolase is a phosphatase. *Proc. Natl. Acad. Sci. U.S.A.* 100 1552–1557. 10.1073/pnas.0437829100 12574508 PMC149870

[B9] Czuba E. Steliga A. Lietzau G. Kowiañski P. (2017). Cholesterol as a modifying agent of the neurovascular unit structure and function under physiological and pathological conditions. *Metab. Brain Dis.* 32 935–948. 10.1007/s11011-017-0015-3 28432486 PMC5504126

[B10] de Chaves E. I. P. Rusiñol A. E. Vance D. E. Campenot R. B. Vance J. E. (1997). Role of lipoproteins in the delivery of lipids to axons during axonal regeneration. *J. Biol. Chem.* 272 30766–30773. 10.1074/jbc.272.49.30766 9388216

[B11] Decker M. Adamska M. Cronin A. Di Giallonardo F. Burgener J. MArowsky A. (2012). EH3 (ABHD9): the first member of a new epoxide hydrolase family with high activity for fatty acid epoxides. *J. Lipid Res.* 53 2038–2045. 10.1194/jlr.M024448 22798687 PMC3435537

[B12] Decker M. Arand M. Cronin A. (2009). Mammalian epoxide hydrolases in xenobiotic metabolism and signalling. *Arch. Toxicol.* 83 297–318. 10.1007/s00204-009-0416-0 19340413

[B13] Dietschy J. M. (2009). Central nervous system: cholesterol turnover, brain development and neurodegeneration. *Biol. Chem.* 390 287–293. 10.1515/BC.2009.035 19166320 PMC3066069

[B14] Enayetallah A. E. French R. A. Thibodeau M. S. Grant D. F. (2004). Distribution of soluble epoxide hydrolase and of cytochrome P450 2C8, 2C9, and 2J2 in human tissues. *J. Histochem. Cytochem.* 52 447–454. 10.1177/002215540405200403 15033996

[B15] Enayetallah A. E. Grant D. F. (2006). Effects of human soluble epoxide hydrolase polymorphisms on isoprenoid phosphate hydrolysis. *Biochem. Biophys. Res. Commun.* 341 254–260. 10.1016/j.bbrc.2005.12.180 16414022

[B16] EnayetAllah A. E. Luria A. Luo B. Tsai H. J. Sura P. Hammock B. D. (2008). Opposite regulation of cholesterol levels by the phosphatase and hydrolase domains of soluble epoxide hydrolase. *J. Biol. Chem.* 283 36592–36598. 10.1074/jbc.M806315200 18974052 PMC2605982

[B17] Farrer L. A. Cupples L. A. Haines J. L. Hyman B. Kukull W. A. Mayeux R. (1997). Effects of age, sex, and ethnicity on the association between apolipoprotein E genotype and Alzheimer disease: a meta-analysis. *JAMA* 278 1349–1356. 10.1001/jama.278.16.1349 9343467

[B18] Fornage M. Boerwinkle E. Doris P. A. Jacobs D. Liu K. Wong N. D. (2004). Polymorphism of the soluble epoxide hydrolase is associated with coronary artery calcification in African-American subjects: the coronary artery risk development in young adults (CARDIA) study. *Circulation* 109 335–339. 10.1161/01.cir.0000109487.46725.02 14732757

[B19] Fretland A. J. Omiecinski C. J. (2000). Epoxide hydrolases: biochemistry and molecular biology. *Chem. Biol. Interact* 129 41–59. 10.1016/s0009-2797(00)00197-6 11154734

[B20] Gamba P. Staurenghi E. Testa G. Giannelli S. Sottero B. Leonarduzzi G. (2019). A crosstalk between brain cholesterol oxidation and glucose metabolism in Alzheimer’s disease. *Front. Neurosci.* 13:556. 10.3389/fnins.2019.00556 31213973 PMC6554318

[B21] Genaro-Mattos T. C. Anderson A. Allen L. B. Korade Z. Mirnics K. (2019). Cholesterol biosynthesis and uptake in developing neurons. *ACS Chem. Neurosci.* 10 3671–3681. 10.1021/acschemneuro.9b00248 31244054 PMC7184320

[B22] Grimm M. O. Grimm H. S. Tomic I. Beyreuther K. Hartmann T. Bergmann C. (2008). Independent inhibition of Alzheimer disease β-and γ-secretase cleavage by lowered cholesterol levels. *J. Biol. Chem.* 283 11302–11311. 10.1074/jbc.m801520200 18308724

[B23] Harris T. R. Aronov P. A. Hammock B. D. (2008). Soluble epoxide hydrolase homologs in Strongylocentrotus purpuratus suggest a gene duplication event and subsequent divergence. *DNA Cell Biol.* 27 467–477. 10.1089/dna.2008.0751 18554159 PMC2858585

[B24] Harris T. R. Hammock B. D. (2013). Soluble epoxide hydrolase: gene structure, expression and deletion. *Gene* 526 61–74. 10.1016/j.gene.2013.05.008 23701967 PMC3733540

[B25] He J. Wang C. Zhu Y. Ai D. (2016). Soluble epoxide hydrolase: a potential target for metabolic diseases:. *J. Diabetes* 8 305–313. 10.1111/1753-0407.12358 26621325

[B26] Helzner E. P. Luchsinger J. A. Scarmeas N. Cosentino S. Brickman A. M. Glymour M. M. (2009). Contribution of vascular risk factors to the progression in Alzheimer disease. *Arch. Neurol.* 66 343–348. 10.1001/archneur.66.3.343 19273753 PMC3105324

[B27] Holtzman D. M. Herz J. Bu G. (2012). Apolipoprotein E and apolipoprotein E receptors: normal biology and roles in Alzheimer disease. *Cold Spring Harb. Perspect. Med.* 2:a006312. 10.1101/cshperspect.a006312 22393530 PMC3282491

[B28] Hou H.-H. Hammock B. D. Su K. H. Morisseau C. Kou Y. R. Imaoka S. (2011). N-terminal domain of soluble epoxide hydrolase negatively regulates the VEGF-mediated activation of endothelial nitric oxide synthase. *Cardiovasc. Res.* 93 120–129. 10.1093/cvr/cvr267 22072631 PMC3243038

[B29] Hou H.-H. Liao Y. J. Hsiao S. H. Shyue S. K. Lee T. S. (2015). Role of phosphatase activity of soluble epoxide hydrolase in regulating simvastatin-activated endothelial nitric oxide synthase. *Sci. Rep.* 5:13524. 10.1038/srep13524 26304753 PMC4548251

[B30] Huang H.-J. Wang Y.-T. Lin H.-C. Lee Y.-H. Lin A. M.-Y. (2018). Soluble epoxide hydrolase inhibition attenuates MPTP-induced neurotoxicity in the nigrostriatal dopaminergic system: involvement of α-synuclein aggregation and ER stress. *Mol. Neurobiol.* 55 138–144. 10.1007/s12035-017-0726-9 28822080

[B31] Imig J. D. (2013). Epoxyeicosatrienoic acids, 20-hydroxyeicosatetraenoic acid, and renal microvascular function. *Prostaglandins Other Lipid Mediat.* 104 2–7. 10.1016/j.prostaglandins.2013.01.002 23333581 PMC3664103

[B32] Imig J. D. Hammock B. D. (2009). Soluble epoxide hydrolase as a therapeutic target for cardiovascular diseases. *Nat. Rev. Drug Discov.* 8 794–805. 10.1038/nrd2875 19794443 PMC3021468

[B33] Iyer A. Kauter K. Alam M. A. Hwang S. H. Morisseau C. Hammock B. D. (2011). Pharmacological inhibition of soluble epoxide hydrolase ameliorates diet-induced metabolic syndrome in rats. *Exp. Diabetes Res.* 2012:758614. 10.1155/2012/758614 22007192 PMC3191770

[B34] Jafari R. M. Ghahremani M. H. Rahimi N. Shadboorestan A. Rashidian A. Esmaeili J. (2018). The anticonvulsant activity and cerebral protection of chronic lithium chloride via NMDA receptor/nitric oxide and phospho-ERK. *Brain Res. Bull.* 137 1–9. 10.1016/j.brainresbull.2017.10.015 29102713

[B35] Jiang X. Guo M. Su J. Lu B. Ma D. Zhang R. (2012). Simvastatin blocks blood-brain barrier disruptions induced by elevated cholesterol both in vivo and in vitro. *Int. J. Alzheimer’s Dis.* 2012:109324. 10.1155/2012/109324 22506129 PMC3296225

[B36] Junier M. P. Dray F. Blair I. Capdevila J. Dishman E. Falck J. R. (1990). Epoxygenase products of arachidonic acid are endogenous constituents of the hypothalamus involved in D2 receptor-mediated, dopamine-induced release of somatostatin. *Endocrinology.* 126 1534–1540. 10.1210/endo-126-3-1534 1968382

[B37] Kramer J. Proschak E. (2017). Phosphatase activity of soluble epoxide hydrolase. *Prostaglandins Other Lipid Mediat.* 133 88–92. 10.1016/j.prostaglandins.2017.07.002 28729091

[B38] Kramer J. S. Woltersdorf S. Duflot T. Hiesinger K. Lillich F. F. Knöll F. (2019). Discovery of the first in vivo active inhibitors of the soluble epoxide hydrolase phosphatase domain. *J. Med. Chem.* 62 8443–8460. 10.1021/acs.jmedchem.9b00445 31436984 PMC7262789

[B39] Lee C.-H. Olson P. Evans R. M. (2003). Minireview: lipid metabolism, metabolic diseases, and peroxisome proliferator-activated receptors. *Endocrinology* 144 2201–2207. 10.1210/en.2003-0288 12746275

[B40] Lee C. R. North K. E. Bray M. S. Fornage M. Seubert J. M. Newman J. W. (2006). Genetic variation in soluble epoxide hydrolase (EPHX2) and risk of coronary heart disease: the Atherosclerosis Risk in Communities (ARIC) study. *Hum. Mol. Genet.* 15 1640–1649. 10.1093/hmg/ddl085 16595607 PMC2040335

[B41] Lin M.-E. Herr D. R. Chun J. (2010). Lysophosphatidic acid (LPA) receptors: signaling properties and disease relevance. *Prostaglandins Other Lipid Mediat.* 91 130–138. 10.1016/j.prostaglandins.2009.02.002 20331961 PMC2845529

[B42] Loera-Valencia R. Goikolea J. Parrado-Fernandez C. Merino-Serrais P. Maioli S. (2019). Alterations in cholesterol metabolism as a risk factor for developing Alzheimer’s disease: potential novel targets for treatment. *J. Steroid Biochem. Mol. Biol.* 190 104–114. 10.1016/j.jsbmb.2019.03.003 30878503

[B43] Lund E. G. Xie C. Kotti T. Turley S. D. Dietschy J. M. Russell D. W. (2003). Knockout of the cholesterol 24-hydroxylase gene in mice reveals a brain-specific mechanism of cholesterol turnover. *J. Biol. Chem.* 278 22980–22988. 10.1074/jbc.m303415200 12686551

[B44] Luria A. Morisseau C. Tsai H. J. Yang J. Inceoglu B. De Taeye B. (2009). Alteration in plasma testosterone levels in male mice lacking soluble epoxide hydrolase. *Am. J. Physiol. -Endocrinol. Metab.* 297 E375–E383. 10.1152/ajpendo.00131.2009 19458064 PMC2724109

[B45] Lütjohann D. Breuer O. Ahlborg G. Nennesmo I. Sidén A. Diczfalusy U. (1996). Cholesterol homeostasis in human brain: evidence for an age-dependent flux of 24S-hydroxycholesterol from the brain into the circulation. *Proc. Natl. Acad. Sci. U.S.A.* 93 9799–9804. 10.1073/pnas.93.18.9799 8790411 PMC38509

[B46] Mahley R. W. Rall S. C. Jr. (2000). Apolipoprotein E: far more than a lipid transport protein. *Annu. Rev. Genomics Hum. Genet.* 1 507–537. 10.1146/annurev.genom.1.1.507 11701639

[B47] Mangels N. Awwad K. Wettenmann A. Dos Santos L. R. Frömel T. Fleming I. (2016). The soluble epoxide hydrolase determines cholesterol homeostasis by regulating AMPK and SREBP activity. *Prostaglandins Other Lipid Mediat.* 125 30–39. 10.1016/j.prostaglandins.2016.05.003 27179554

[B48] Marowsky A. Burgener J. Falck J. R. Fritschy J. M. Arand M. (2009). Distribution of soluble and microsomal epoxide hydrolase in the mouse brain and its contribution to cerebral epoxyeicosatrienoic acid metabolism. *Neuroscience* 163 646–661. 10.1016/j.neuroscience.2009.06.033 19540314

[B49] Masters C. L. Bateman R. Blennow K. Rowe C. C. Sperling R. A. Cummings J. L. (2015). Alzheimer’s disease. *Nat. Rev. Dis. Primers* 1:15056. 10.1038/nrdp.2015.56 27188934

[B50] Mauch D. H. Nägler K. Schumacher S. Göritz C. Müller E. C. Otto A. (2001). CNS synaptogenesis promoted by glia-derived cholesterol. *Science* 294 1354–1357. 10.1126/science.294.5545.1354 11701931

[B51] Morisseau C. Hammock B. D. (2013). Impact of soluble epoxide hydrolase and epoxyeicosanoids on human health. *Annu. Rev. Pharmacol. Toxicol.* 53 37–58. 10.1146/annurev-pharmtox-011112-140244 23020295 PMC3578707

[B52] Morisseau C. Sahdeo S. Cortopassi G. Hammock B. D. (2013). Development of an HTS assay for EPHX2 phosphatase activity and screening of nontargeted libraries. *Anal. Biochem.* 434 105–111. 10.1016/j.ab.2012.11.017 23219563 PMC3557602

[B53] Morisseau C. Schebb N. H. Dong H. Ulu A. Aronov P. A. Hammock B. D. (2012). Role of soluble epoxide hydrolase phosphatase activity in the metabolism of lysophosphatidic acids. *Biochem. Biophys. Res. Commun.* 419 796–800. 10.1016/j.bbrc.2012.02.108 22387545 PMC3313618

[B54] Morris A. Panchatcharam M. Cheng H. Y. Federico L. Fulkerson Z. Selim S. (2009). Regulation of blood and vascular cell function by bioactive lysophospholipids. *J. Thromb. Haemost.* 7 38–43. 10.1111/j.1538-7836.2009.03405.x 19630765 PMC2801156

[B55] Negro-Vilar A. Snyder G. D. Falck J. R. Manna S. Chacos N. Capdevila J. (1985). Involvement of eicosanoids in release of oxytocin and vasopressin from the neural lobe of the rat pituitary. *Endocrinology* 116 2663–2668. 10.1210/endo-116-6-2663 3922746

[B56] Newman J. W. Morisseau C. Hammock B. D. (2005). Epoxide hydrolases: their roles and interactions with lipid metabolism. *Progr. Lipid Res.* 44 1–51. 10.1016/j.plipres.2004.10.001 15748653

[B57] Oguro A. Sakamoto K. Suzuki S. Imaoka S. (2009). Contribution of hydrolase and phosphatase domains in soluble epoxide hydrolase to vascular endothelial growth factor expression and cell growth. *Biol. Pharm. Bull.* 32 1962–1967. 10.1248/bpb.32.1962 19952412

[B58] Pardeshi R. Bolshette N. Gadhave K. Arfeen M. Ahmed S. Jamwal R. (2019). Docosahexaenoic acid increases the potency of soluble epoxide hydrolase inhibitor in alleviating streptozotocin-induced Alzheimer’s disease-like complications of diabetes. *Front. Pharmacol.* 10:288. 10.3389/fphar.2019.00288 31068802 PMC6491817

[B59] Petrov A. Kasimov M. R. Zefirov A. L. (2016). Brain cholesterol metabolism and its defects: linkage to neurodegenerative diseases and synaptic dysfunction. *Acta Natu.* 8 58–73. 10.32607/20758251-2016-8-1-58-73 27099785 PMC4837572

[B60] Petrov A. Kasimov M. R. Zefirov A. L. (2017). Cholesterol in the pathogenesis of Alzheimer’s, Parkinson’s diseases and autism: link to synaptic dysfunction. *Acta Nat.* 9 26–37. 10.32607/20758251-2017-9-1-26-37 28461971 PMC5406657

[B61] Popp J. Meichsner S. Kölsch H. Lewczuk P. Maier W. Kornhuber J. (2013). Cerebral and extracerebral cholesterol metabolism and CSF markers of Alzheimer’s disease. *Biochem. Pharmacol.* 86 37–42. 10.1016/j.bcp.2012.12.007 23291240

[B62] Przybyla-Zawislak B. D. Srivastava P. K. Vazquez-Matias J. Mohrenweiser H. W. Maxwell J. E. Hammock B. D. (2003). Polymorphisms in human soluble epoxide hydrolase. *Mol. Pharmacol.* 64 482–490. 12869654 10.1124/mol.64.2.482

[B63] Riccio A. Alvania R. S. Lonze B. E. Ramanan N. Kim T. Huang Y. (2006). A nitric oxide signaling pathway controls CREB-mediated gene expression in neurons. *Mol. Cell.* 21 283–294. 10.1016/j.molcel.2005.12.006 16427017

[B64] Sato K. Emi M. Ezura Y. Fujita Y. Takada D. Ishigami T. (2004). Soluble epoxide hydrolase variant (Glu287Arg) modifies plasma total cholesterol and triglyceride phenotype in familial hypercholesterolemia: intrafamilial association study in an eight-generation hyperlipidemic kindred. *J. Hum. Genet.* 49 29–34. 10.1007/s10038-003-0103-6 14673705

[B65] Schulz E. Jansen T. Wenzel P. Daiber A. Münzel T. (2008). Nitric oxide, tetrahydrobiopterin, oxidative stress, and endothelial dysfunction in hypertension. *Antioxid. Redox Signal.* 10 1115–1126. 10.1089/ars.2007.1989 18321209

[B66] Schuman E. M. Madison D. V. (1991). A requirement for the intercellular messenger nitric oxide in long-term potentiation. *Science* 254 1503–1506. 10.1126/science.1720572 1720572

[B67] Sciorra V. A. Morris A. J. (2002). Roles for lipid phosphate phosphatases in regulation of cellular signaling. *Biochim. Biophys. Acta)Mol. Cell Biol. Lipids* 1582 45–51. 10.1016/s1388-1981(02)00136-1 12069809

[B68] Shen H. C. Hammock B. D. (2012). Discovery of inhibitors of soluble epoxide hydrolase: a target with multiple potential therapeutic indications. *J. Med. Chem.* 55 1789–1808. 10.1021/jm201468j 22168898 PMC3420824

[B69] Sura P. Sura R. Enayetallah A. E. Grant D. F. (2008). Distribution and expression of soluble epoxide hydrolase in human brain. *J. Histochem. Cytochem.* 56 551–559. 10.1369/jhc.2008.950659 18319271 PMC2386770

[B70] Taguchi N. Nakayama S. Tanaka M. (2016). Single administration of soluble epoxide hydrolase inhibitor suppresses neuroinflammation and improves neuronal damage after cardiac arrest in mice. *Neurosci. Res.* 111 56–63. 10.1016/j.neures.2016.05.002 27184295

[B71] Terashvili M. Tseng L. F. Wu H. E. Narayanan J. Hart L. M. Falck J. R. (2008). Antinociception produced by 14,15-epoxyeicosatrienoic acid is mediated by the activation of beta-endorphin and met-enkephalin in the rat ventrolateral periaqueductal gray. *J. Pharmacol. Exp. Ther.* 326 614–622. 10.1124/jpet.108.136739 18492947 PMC2567871

[B72] Thomson S. J. Askari A. Bishop-Bailey D. (2012). Anti-inflammatory effects of epoxyeicosatrienoic acids. *Int. J. o Vasc. Med.* 2012:605101. 10.1155/2012/605101 22848834 PMC3405717

[B73] Viola K. L. Klein W. L. (2015). Amyloid β oligomers in Alzheimer’s disease pathogenesis, treatment, and diagnosis. *Acta Neuropathol.* 129 183–206. 10.1007/s00401-015-1386-3 25604547 PMC4390393

[B74] Wei Q. Doris P. A. Pollizotto M. V. Boerwinkle E. Jacobs D. R. Jr. Siscovick D. S. (2007). Sequence variation in the soluble epoxide hydrolase gene and subclinical coronary atherosclerosis: interaction with cigarette smoking. *Atherosclerosis* 190 26–34. 10.1016/j.atherosclerosis.2006.02.021 16545818

[B75] Wu H.-F. Yen H. J. Huang C. C. Lee Y. C. Wu S. Z. Lee T. S. (2015). Soluble epoxide hydrolase inhibitor enhances synaptic neurotransmission and plasticity in mouse prefrontal cortex. *J. Biomed. Sci.* 22:94. 10.1186/s12929-015-0202-7 26494028 PMC4618874

[B76] Wu Q. Cai H. Song J. Chang Q. (2017). The effects of sEH inhibitor on depression-like behavior and neurogenesis in male mice. *J. Neurosci. Res.* 95 2483–2492. 10.1002/jnr.24080 28699310

[B77] Wu Q. J. Tymianski M. (2018). Targeting NMDA receptors in stroke: new hope in neuroprotection. *Mol. Brain* 11:15. 10.1186/s13041-018-0357-8 29534733 PMC5851248

[B78] Xie Y. Tan Y. Zheng Y. Du X. Liu Q. (2017). Ebselen ameliorates β-amyloid pathology, tau pathology, and cognitive impairment in triple-transgenic Alzheimer’s disease mice. *JBIC J. Biol. Inorg. Chem.* 22 851–865. 10.1007/s00775-017-1463-2 28502066

[B79] Xue-shan Z. Juan P. Qi W. Zhong R. Li-Hong P. Zhi-Han T. (2016). Imbalanced cholesterol metabolism in Alzheimer’s disease. *Clin. Chim. Acta* 456 107–114. 10.1016/j.cca.2016.02.024 26944571

[B80] Yu Z. Xu F. Huse L. M. Morisseau C. Draper A. J. Newman J. W. (2000). Soluble epoxide hydrolase regulates hydrolysis of vasoactive epoxyeicosatrienoic acids. *Circ. Res.* 87 992–998. 10.1161/01.res.87.11.992 11090543

[B81] Yuan L. Liu J. Dong R. Zhu J. Tao C. Zheng R. (2016). 14, 15-epoxyeicosatrienoic acid promotes production of brain derived neurotrophic factor from astrocytes and exerts neuroprotective effects during ischaemic injury. *Neuropathol. Appl. Neurobiol.* 42 607–620. 10.1111/nan.12291 26526810

[B82] Zhang J. Liu Q. (2015). Cholesterol metabolism and homeostasis in the brain. *Protein Cell* 6 254–264. 10.1007/s13238-014-0131-3 25682154 PMC4383754

